# Connexins, Cell Proliferation and Second Messengers in the Crystalline Lens

**Published:** 2012-01

**Authors:** Thomas W White

**Affiliations:** Department of Physiology and Biophysics, Stony Brook University School of Medicine, Stony Brook, New York, USA

Gap junctions are responsible for the direct coupling of cells that enables intercellular exchange of ions, small metabolites and second messengers. Lens epithelial cells are well coupled (Fig. 1a) by connexin (Cx) channels that constitute the structural subunits of gap junctions.^1^,^2^ Surprisingly, epithelial cell proliferation during the early postnatal period (Fig. 1b) was found to be profoundly influenced by the type of connexin subunit present in the gap junction channels between cells. The loss, or functional replacement, of Cx50 caused a significant decrease in the number of dividing cells during the first postnatal week, suggesting that Cx50-mediated communication was essential for peak mitosis to occur.^3^,^4^ In turn, the magnitude of coupling mediated by specific lens connexins can be differentially modulated by the same mitogenic signaling pathways that stimulate mitosis (Fig. 1c). For example, we have previously shown that the mitogen activated protein kinase (MAPK) pathway specifically modulated Cx50, but not Cx46 *in vitro*, and that Cx50 mediated communication and MAPK signaling interacted in the regulation of lens development and homeostasis *in vivo*.^4^ More recently, we have seen that modulation of phosphoinositide 3-kinase (PI3K) activity also impacted cell proliferation and Cx50 mediated coupling in a similar manner to changes observed after perturbation of MAPK signaling. Integration of these observations has led us to the hypothesis that signal transduction drives mitosis in part through the generation of soluble second messengers that can permeate through gap junction channels, which are coordinately regulated by the same signal transduction pathways.

To further test this hypothesis, we have been developing methods to quantitatively measure the permeation of relevant second messengers such as cAMP through gap junction channels.^5^ The detection of intercellular cAMP transfer was accomplished by co-culturing cell pairs where one cell expressed the connexin of interest, green fluorescent protein (GFP), and the cyclic nucleotide gated channel from sea urchin SpIH, while the other cell expressed only the connexin being studied and red fluorescent protein (RFP, Fig. 1d). cAMP transfer was monitored by recording the activity of the SpIH current while simultaneously measuring junctional conductance using dual patch clamp. For the connexin and GFP containing source cell, the pipette was in whole cell mode and contained 500 μM cAMP. For the recipient cell containing connexin, RFP and SpIH, the pipette was in perforated patch mode. Over time, the SpIH current in the recipient cell increased to a new steady state value due to cAMP diffusion from its source to recipient cell. A quantitative assay for cyclic nucleotide permeation through gap junction channels will allow us to test our hypothesis that lens connexins exhibit intrinsic differences in permeability to second messenger molecules such as cAMP that can influence mitosis. Data derived from this assay will also help to further define how diversity in connexin proteins influences intercellular communication in the lens during normal growth and development.

## Figures and Tables

**Figure 1.**
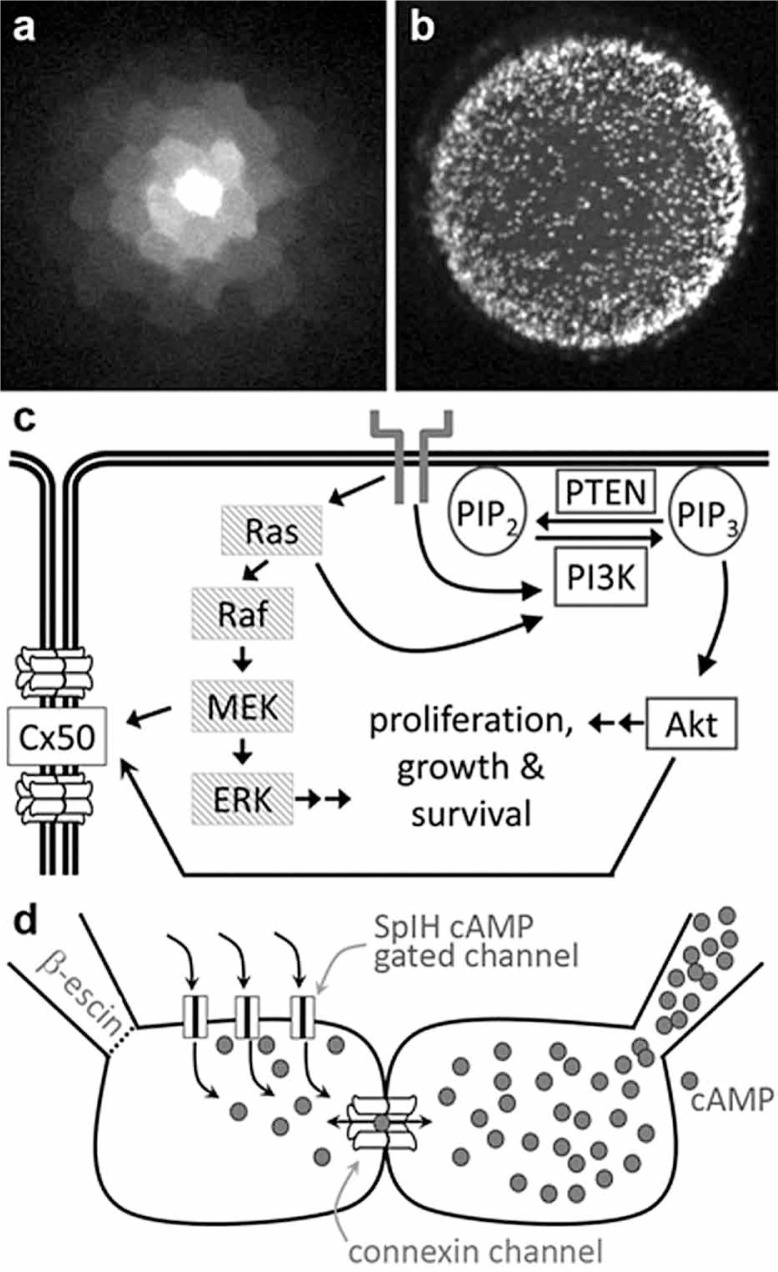
Connexins, cell proliferation and second messengers in the lens. (a) Lens epithelial cells pass small fluorescent dyes through an extensive network of gap junction channels. (b) During early postnatal growth, mitotic activity is high across the entire epithelium as shown by EdU staining of an intact postnatal day 2 mouse lens. (c) Cx50 interacts with lens signal transduction pathways, including the MAPK and PI3K pathways that stimulate mitosis. (d) Schematic drawing of a cell pair based assay to measure cyclic nucleotide permeability through connexin channels.
